# The effect of cigarillo packaging elements on young adult perceptions of product flavor, taste, smell, and appeal

**DOI:** 10.1371/journal.pone.0196236

**Published:** 2018-04-19

**Authors:** Clare Meernik, Leah M. Ranney, Allison J. Lazard, KyungSu Kim, Tara L. Queen, Aya Avishai, Marcella H. Boynton, Paschal J. Sheeran, Adam O. Goldstein

**Affiliations:** 1 Department of Family Medicine, University of North Carolina at Chapel Hill School of Medicine, Chapel Hill, North Carolina, United States of America; 2 School of Media and Journalism, University of North Carolina at Chapel Hill, Chapel Hill, North Carolina, United States of America; 3 Lineberger Comprehensive Cancer Center, University of North Carolina at Chapel Hill, Chapel Hill, North Carolina, United States of America; 4 Department of Psychology and Neuroscience, University of North Carolina at Chapel Hill, Chapel Hill, North Carolina, United States of America; 5 Department of Health Behavior, Gillings School of Global Public Health, University of North Carolina at Chapel Hill, Chapel Hill, North Carolina, United States of America; University of California, San Diego, UNITED STATES

## Abstract

**Introduction:**

Product packaging has long been used by the tobacco industry to target consumers and manipulate product perceptions. This study examines the extent to which cigarillo packaging influences perceptions of product flavor, taste, smell, and appeal.

**Methods:**

A web-based experiment was conducted among young adults. Participants viewed three randomly selected cigarillo packs, varying on pack flavor descriptor, color, type, branding, and warning—totaling 180 pack images. Mixed-effects models were used to estimate the effect of pack elements on product perceptions.

**Results:**

A total of 2,664 current, ever, and never little cigar and cigarillo users participated. Cigarillo packs with a flavor descriptor were perceived as having a more favorable taste (β = 0.21, p < .001) and smell (β = 0.14, p < .001) compared to packs with no flavor descriptor. Compared to packs with no color, pink and purple packs were more likely to be perceived as containing a flavor (β = 0.11, p < .001), and were rated more favorably on taste (β = 0.17, p < .001), smell (β = 0.15, p < .001), and appeal (β = 0.16, p < .001). While warnings on packs decreased favorable perceptions of product taste (pictorial: β = -0.07, p = .03) and smell (text-only: β = -0.08, p = .01; pictorial: β = -0.09, p = .007), warnings did not moderate the effects of flavor descriptor or color.

**Conclusions:**

To our knowledge, this study provides the first quantitative evidence that cigarillo packaging alters consumers’ cognitive responses, and warnings on packs do not suffice to overcome the effects of product packaging. The findings support efforts at federal, state, and local levels to prohibit flavor descriptors and their associated product flavoring in non-cigarette products such as cigarillos, along with new data that supports restrictions on flavor cues and colors.

## Introduction

As policies on tobacco marketing have become increasingly restrictive, the tobacco industry has focused on packaging to promote their products [1–4] and manipulate consumers’ perceptions of products [5,6]. Cigarette pack design elements such as descriptor terms (e.g., silver, smooth, natural), pack color, flavor descriptors, and pack shape are associated with reduced product harm perceptions and increased product appeal, purchase interest, and consumption [6–13].

Ways to decrease cigarette packaging influence have included warnings and plain packaging. Warnings on packs can both counteract appealing pack design elements and communicate health messages to consumers. Compared to text-only warnings, pictorial warnings on cigarette packs attract more attention, evoke more negative affect and attitudes towards the product, and more effectively deter initiation and promote intentions to quit [14]. Pictorial warnings increase forgoing cigarettes, quit attempts, and successful cessation [15]. Plain packaging is also increasingly being implemented around the world to reduce the appeal of tobacco products by removing brand imagery and attractive colors and fonts from packages [16]. Cigarette packs with plain packaging are perceived as less appealing, less satisfying, and rated as having lower taste and quality [17]. Though studies assessing the impact of plain packaging on behavioral outcomes are limited, current evidence suggests that plain packaging may increase quit attempts and calls to quitlines, as well as reduce smoking consumption and prevalence [17].

Although non-cigarette pack elements may have a similar effect as cigarette pack elements on product perceptions and behavior, research on other products—particularly little cigars and cigarillos (LCCs)—is sparse. Findings concerning cigarette packaging effects do not necessarily translate to LCC packaging effects, as LCCs have differing characteristics, including unique pack designs and flavors. LCCs are two of the three major cigar products, with the other being traditional, or large, cigars [18]. LCCs have experienced a rise in popularity in recent years, attributable to factors such as their affordability compared to cigarettes due to lower taxes and the availability of flavored products [19]. Current LCC smokers are more likely to be younger, male, black or Hispanic, lower SES, and use or have tried other tobacco products [20,21]. Further, U.S. adults who are lesbian, gay, or bisexual are more likely to be cigar smokers (i.e., users of cigars, little cigars, or cigarillos) than those who are heterosexual [22].

There is a pressing need for research on LCC packaging given the U.S. Food and Drug Administration’s (FDA) extended regulatory authority over all cigars, including LCCs [23], and the FDA’s recently issued Advance Notice of Proposed Rulemaking (ANPRM) to seek comment on the role of flavors in tobacco products [24]. The FDA proposed deeming rule did include a ban on characterizing flavors in newly deemed products (e.g., LCCs), but that proposed ban was deleted in the final ruling by the Office of Management and Budget [25]. Although the issuing of the ANPRM does not guarantee future regulatory action on flavored tobacco products, the FDA is taking action to gather more evidence on flavored tobacco products and their role in product appeal and use.

Understanding the effect of LCC pack elements that directly or indirectly suggest product flavor is particularly relevant, as flavored tobacco products are perceived as less harmful and flavors influence tobacco product experimentation, initiation, and continuation of use [26,27]. Qualitative evidence from youth and young adults finds that flavors are the primary appeal of LCC packaging [28]; further, the visual, smell, and taste cues from flavor descriptors and associated colors on packs influence affect toward and use of LCCs [29]. Similarly, survey data from the U.S. and Canada shows that flavors are the primary reason young adults use LCCs [20,30].

However, to date, no studies have experimentally tested consumers’ cognitive response to packaging of LCCs, particularly cigarillos. This study investigated how flavor descriptors, color, type, branding, and warnings on cigarillo packs each influence perceptions of flavor, taste, smell, and appeal among young adults—the age group most likely to be using LCCs [20,21].

## Methods

### Participants and procedures

Young adults were recruited between February and March 2017 through Amazon Mechanical Turk (MTurk), an online crowdsourcing tool through which individuals earn small amounts of money by completing online tasks. MTurk is increasingly being used for social science and health research to cost-effectively recruit large and diverse samples with valid results [31–33]. Study eligibility included age (18–26 years old) and U.S. residence; current (past 30-day use), ever, and never LCC users were included.

After providing written informed consent, participants responded to survey items regarding pre-existing perceptions of LCCs. Participants then viewed three randomly selected, manipulated cigarillo pack images one at a time and answered outcome measures after each image (see Measures section). Participants were paid $2.35 through Amazon MTurk for completing the entire survey; median survey duration was 10.8 minutes The University of North Carolina at Chapel Hill Institutional Review Board approved this study (# 16–0335).

### Stimuli

**Fig 1:**
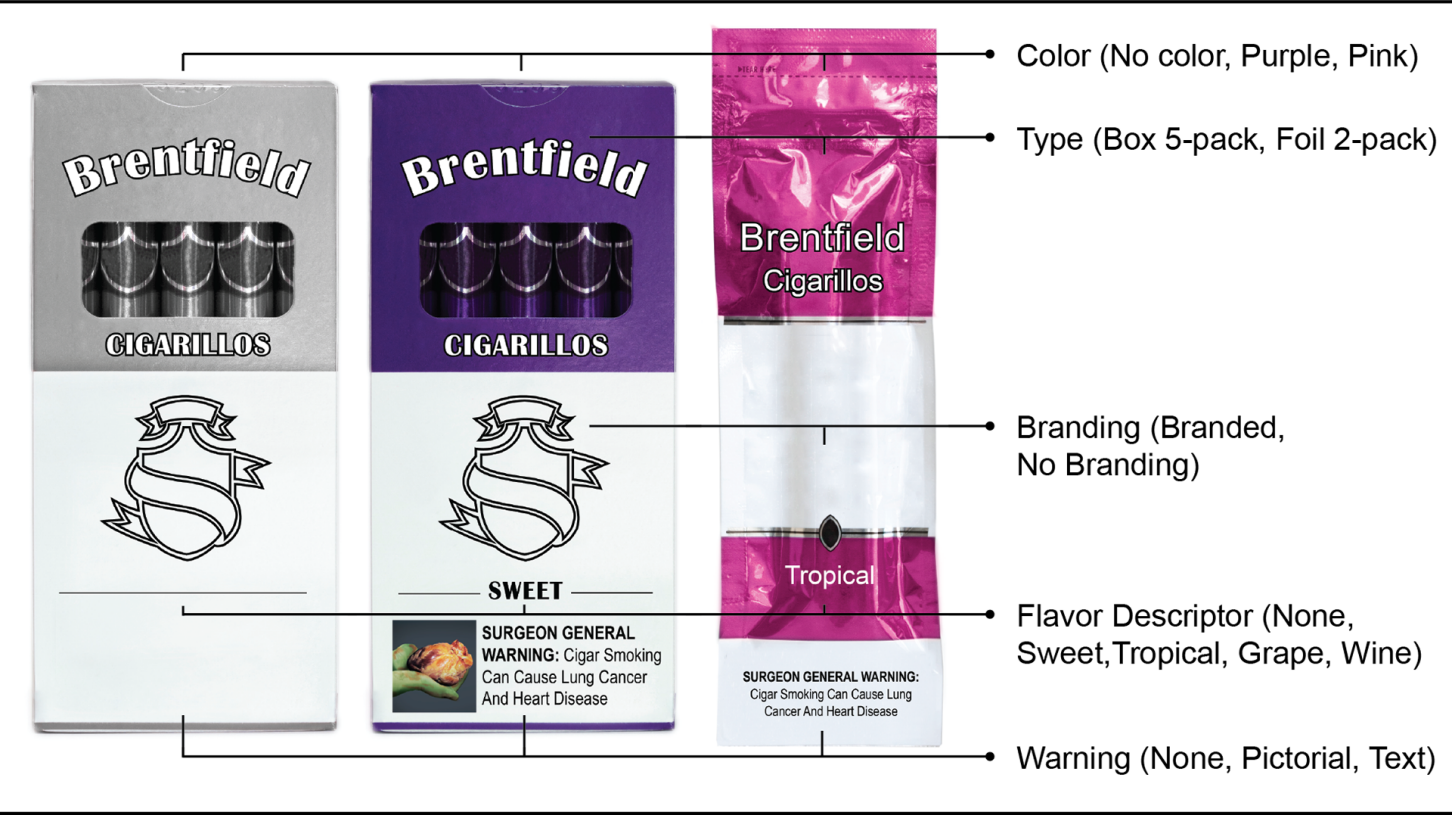
Cigarillo pack manipulations.

Cigarillo packs were branded with a fictitious name, “Brentfield”, a brand successfully used in prior research [34,35] to minimize the influence of brand loyalty and pre-existing brand perceptions. Cigarillo packs were manipulated on five elements, generating 180 pack images: 1) flavor descriptor (none, Sweet, Grape, Wine, Tropical); 2) pack color (no color, pink, purple); 3) pack type (box 5-pack, foil 2-pack); 4) branding (no branding, branded); 5) warning (no warning, text-only, pictorial) (Fig 1). Variations of each element were based on common variations in the market. For instance, fruit (e.g., Grape), Sweet, and Wine hold the top market share among flavored cigar products (which include LCCs and large cigars) [36,37]. Tropical was also chosen as a flavor descriptor in order to represent the rising popularity of “other”, non-descript flavors [37]. The purple and pink pack colors were selected based on their ability to realistically represent any of the four flavor descriptors (i.e., each color was congruent with respective flavor descriptors). The box 5-pack and the foil 2-pack were chosen because these pack types have a high percentage of the market share [36]. For pack branding, the no branding condition was used to represent a pack similar to one with plain packaging (e.g., no stylized font or brand logos). Lastly, warnings covered approximately 25% of the bottom of the pack, with cigar-specific mandated warning text (which also applies to cigarillos) [38] rated to be highly believable among U.S. adults—“WARNING: Cigar Smoking Can Cause Lung Cancer and Heart Disease” [39]; the pictorial warning showed a diseased heart.

### Measures

LCC use was assessed through two items: 1) ever use of little cigars or cigarillos, even one or two puffs and 2) days smoked LCCs in the past 30 days [40]. Current use was defined as any use in the past 30 days. Quotas for the study were set at 1,100 each for never, ever, and current LCC users. Quotas for never and current LCC users were reached, but the quota for ever LCC users was not fully met before the survey closed due to time constraints (n = 909 ever LCC users).

Table 1 details other covariate and outcome measures. Pre-existing perception measures were asked prior to experimental manipulations. Cronbach’s alpha was calculated for related sets of survey items that were grouped as one variable (i.e., attitudes, risk of addiction, and risk of health problems); all showed sufficiently high reliability (Table 1). Additionally, the following sociodemographic characteristics were assessed: age, gender (male, female, other), race (white, black or African-American, Asian, other), ethnicity (Hispanic, non-Hispanic), sexual orientation (heterosexual, lesbian/gay/bisexual, other), education, and parental education (high school or less, some college or associate’s degree, bachelor’s degree or more). As younger participants may not have completed their education yet, highest parental education was used as the education value for participants aged 18–22; own education was used for participants aged 23–26. Past 30-day use of tobacco products other than LCCs (e.g., cigarettes, cigars, hookah, e-cigarettes) was also assessed. Pre-existing perceptions of LCCs, sociodemographic characteristics, and other tobacco use were included as control covariates in all models due to their clinical [20–22] and statistical significance (see S2 Table for unadjusted analyses).

**Table 1:** Covariates and outcome measures.

Construct	Item	Scale	Reliability
**Covariates: Pre-existing perceptions**			
Norms A [41]	Most people who are important to me would approve of my smoking little cigars or cigarillos.	5-point (definitely not to definitely yes)	N/A
Norms B [41]	Of the people you know, how many smoke little cigars or cigarillos?	5-point (0 people to 10 or more people)	N/A
Attitudes [42]	Please select the number between the pairs of words that best describes your thoughts on the following statement: I think smoking little cigars or cigarillos is: 1) bad/good; 2) unhealthy/healthy; 3) harmful/harmless; 4) unpleasant/pleasant; 5) not enjoyable/enjoyable; 6) not satisfying/satisfying	7-point	α = 0.90
Risk of addiction [43]	If you smoke little cigars or cigarillos regularly, how likely is it that you would get addicted at some point in the future?	4-point (very unlikely to very likely)	α = 0.83
	If you smoke little cigars or cigarillos regularly, how fearful would you be of getting addicted in the future?	4-point (not at all fearful to very fearful)	
	If you smoke little cigars or cigarillos regularly, how much do you agree or disagree with the following statement: I would feel very vulnerable to addiction.	4-point (strongly disagree to strongly agree)	
Risk of health problems [43]	If you smoke little cigars or cigarillos regularly, how likely is it that you would get health problems at some point in the future?	4-point (very unlikely to very likely)	α = 0.84
	If you smoke little cigars or cigarillos regularly, how fearful would you be of getting health problems in the future?	4-point (not at all fearful to very fearful)	
	If you smoke little cigars or cigarillos regularly, how much do you agree or disagree with the following statement: I would feel very vulnerable to health problems.	4-point (strongly disagree to strongly agree)	
Prototype A [44]	How likeable or dislikeable do you think the type of person who smokes little cigars or cigarillos is?	5-point (very dislikeable to very likeable)	N/A
Prototype B [44]	In general, how similar do you think you are to the type of person who smokes little cigars or cigarillos?	5-point (not at all similar to very similar)	N/A
**Outcomes: Product perceptions**			
Flavor	Do you think these cigarillos contain a flavor other than tobacco?	5-point (definitely not to definitely yes)	N/A
Taste [45,46]	The taste of these cigarillos would: 1) be harsh; 2) be sweet; 3) be fruity; 4) have a tobacco taste	7-point (strongly disagree to strongly agree)	α = 0.75–0.78
		Harsh and tobacco taste items reverse scored (higher score indicates more favorable taste perception)	
Smell [45,46]	The smell of these cigarillos would: 1) be harsh; 2) be sweet; 3) be fruity; 4) have a tobacco smell	7-point (strongly disagree to strongly agree)	α = 0.77–0.79
		Harsh and tobacco smell items reverse scored (higher score indicates more favorable smell perception)	
Appeal [47,48]	The cigarillo pack in this picture is: 1) hip; 2) trendy; 3) appealing	7-point (strongly disagree to strongly agree)	α = 0.93

After answering pre-existing perception questions, participants were shown three randomly selected cigarillo pack images one at a time and responded to all outcome measures after each image. Pack images were randomized through the survey software, with participants having a chance to see any of the 180 pack images for each of their three stimuli. Participants were not assigned to a certain condition (e.g., Sweet flavor, black and white, foil pack, branded, with a text-only warning). It is possible that participants viewed one or more of the same pack images by chance. Outcome measures assessed perceptions of flavor, taste, and smell, and product appeal; measures were developed based on published literature and tobacco industry research. Cronbach’s alpha was calculated for related sets of survey items that were grouped as one outcome variable (i.e., taste, smell, and appeal); all showed sufficiently high reliability (Table 1).

To bolster data quality, three attention check items were randomly placed throughout the survey (e.g., “How often have you died from smoking cigarettes and were not resuscitated? Please select ‘Never’ as your answer to let us know that you read all of the survey instructions.”). Any participant who failed at least one attention check was removed from analysis [49].

### Statistical analysis

The study involved a 5 (flavor descriptor) x 3 (color) x 2 (type) x 2 (branding) x 3 (warning) fractional factorial design that systematically varied cigarillo pack images on five cigarillo packaging elements, yielding 180 unique stimuli. As each participant rated three randomly assigned pack images, this afforded approximately 44 observations per image, also making this a within-between (i.e., mixed) design. Least square means were calculated for the main covariates (i.e., packaging elements and LCC use) and interactions of interest for each of the four outcome variables.

To account for within-individual correlations, linear mixed-effects models with maximum likelihood estimation were used that treated participants as a random effect [50]. Unadjusted and adjusted main effects were estimated for the five cigarillo packaging elements and LCC use. All cigarillo flavor descriptors and colors showed similar effects, so for ease of interpretation, pack flavor was collapsed into flavor descriptor and no flavor descriptor; color was collapsed into colored (pink or purple) and non-colored pack. To further examine the effects of pack elements, a second set of adjusted models included two-way interactions between LCC use and flavor descriptor and color, and between warning and flavor descriptor and color. All adjusted models included pre-existing perceptions of LCCs, sociodemographic characteristics, and past 30-day use of tobacco products other than LCCs as control covariates. Data were analyzed using SAS version 9.3 (SAS Institute, Inc.) with a two-tailed significance level (p < .05).

## Results

A total of 16,813 participants completed the screener, with 18.2% of those (n = 3,063) meeting eligibility criteria (i.e., ages 18–26 and U.S. residence), without surpassing the quota of 1,100 set for each of the three LCC user groups. Excluding participants who failed one or more attention checks, the final sample comprised 2,664 LCC current users (37.1%), ever users (28.5%), and never users (34.4%), with a mean (SD) age of 23.5 (2.0). The majority of participants were female (57%), non-Hispanic (89%), white (76%), heterosexual (80%), and had at least a bachelor’s degree (54%) (Table 2).

**Table 2:** Sample characteristics, n = 2,664.

Characteristic		n or M	% or SD
**Age** (range: 18–26 years old)		23.5	2.0
**Gender**	Male	1095	41.1%
	Female	1527	57.3%
	Other^a^	42	1.6%
**Ethnicity**	Hispanic	282	10.6%
	Non-Hispanic	2382	89.4%
**Race**^**b**^	White	2015	75.6%
	Black or African American	201	7.6%
	Asian	199	7.5%
	Other^c^	248	9.3%
**Sexual orientation**	Heterosexual	2142	80.4%
	Lesbian, gay, or bisexual	450	16.9%
	Other	72	2.8%
**Education**^**d**^	High school or less	358	13.4%
	Some college or associate’s degree	872	32.8%
	Bachelor’s degree or more	1430	53.8%
**LCC use**	Current user	989	37.1%
	Ever user	759	28.5%
	Never user	916	34.4%
**Past 30-day use of tobacco products other than LCCs**	Yes	1207	45.3%
	No	1457	54.7%

^a^Other gender includes transgender and other

^b^One participant with missing data

^c^Other race includes Native Hawaiian/Pacific Islander, American Indian/Alaska Native, other, and multiracial

^d^For participants aged 18–22, education is defined as highest parental education; for participants aged 23–26, education is defined as participants’ highest education; 4 participants with missing data

### Main effects of pack elements

Least square means and unadjusted mixed-effects analyses are reported in S1 Table and S2 Table, respectively, for reference. Unadjusted and adjusted analyses resulted in similar findings. Table 3 presents results from adjusted mixed-effects models. Compared to cigarillo packs with no flavor descriptor, cigarillo packs with any flavor descriptor were more likely to be perceived as containing a flavor (β = 0.36, p < .001), and were rated more favorably on product taste (β = 0.21, p < .001) and smell (β = 0.14, p < .001). Similarly, compared to non-colored packs, pink or purple packs were more likely to be perceived as containing a flavor (β = 0.11, p < .001) and were rated more favorably on product taste (β = 0.17, p < .001), smell (β = 0.15, p < .001), and appeal (β = 0.16, p < .001). Warnings on the cigarillo pack resulted in less positive product perceptions compared to cigarillo packs with no warning; specifically, cigarillo packs with a pictorial warning were perceived as having a less favorable taste (β = -0.07, p = .03), and packs with text-only or pictorial warnings were perceived as having a less favorable smell (β = -0.08, p = .01; β = -0.09, p = .007, respectively). Foil 2-packs were rated as more appealing than box 5-packs (β = 0.19, p < .001). No significant effects were found for branding. Compared to never LCC users, ever users were more likely to perceive packs as containing a flavor (β = 0.14, p < .001) and rated product taste (β = 0.09, p = .03) and smell more favorably (β = 0.18, p < .001), while current users rated product smell more favorably (β = 0.12, p = .04) and rated packs as more appealing (β = 0.20, p = .02).

**Table 3:** Adjusted mixed-effects model results for pack perceptions.

Independent variables	Flavor	Taste	Smell	Appeal
	β (SE)	β (SE)	β (SE)	β (SE)
**Models with only main effects**^**a**^				
**Flavor descriptor**				
None	Ref	Ref	Ref	Ref
Flavor descriptor	0.36 (0.03)^f^	0.21 (0.03)^f^	0.14 (0.03)^f^	0.06 (0.04)
**Color**				
No color	Ref	Ref	Ref	Ref
Pink or purple	0.11 (0.03)^f^	0.17 (0.03)^f^	0.15 (0.03)^f^	0.16 (0.04)^f^
**Type**				
Box 5-pack	Ref	Ref	Ref	Ref
Foil 2-pack	0.05 (0.02)	-0.02 (0.03)	0.003 (0.03)	0.19 (0.03)^f^
**Branding**				
No branding	Ref	Ref	Ref	Ref
Branded	0.01 (0.02)	0.002 (0.03)	0.01 (0.03)	0.02 (0.03)
**Warning**				
No warning	Ref	Ref	Ref	Ref
Text-only	-0.04 (0.03)	-0.06 (0.03)	-0.08 (0.03)^d^	-0.03 (0.04)
Pictorial	-0.02 (0.03)	-0.07 (0.03)^d^	-0.09 (0.03)^e^	-0.01 (0.04)
**LCC use**^**b**^				
Never	Ref	Ref	Ref	Ref
Ever	0.14 (0.04)^f^	0.09 (0.04)^d^	0.18 (0.05)^f^	0.06 (0.06)
Current	0.06 (0.05)	0.04 (0.05)	0.12 (0.06)^d^	0.20 (0.08)^d^
**Models including interaction effects**^**c**^				
**LCC use x Flavor descriptor**				
Current user x Flavor descriptor	-0.22 (0.07)^e^	-0.22 (0.08)^e^	-0.13 (0.08)	0.20 (0.10)^d^
Ever user x Flavor descriptor	-0.08 (0.08)	-0.17 (0.08)^d^	-0.08 (0.08)	0.01 (0.11)
**LCC use x Color**				
Current user x Pink or purple pack	0.05 (0.06)	0.07 (0.06)	0.08 (0.07)	0.07 (0.08)
Ever user x Pink or purple pack	-0.09 (0.07)	0.02 (0.07)	-0.02 (0.07)	-0.10 (0.09)
**Warning x Flavor descriptor**				
Text x Flavor descriptor	0.10 (0.08)	0.12 (0.08)	0.12 (0.08)	0.07 (0.10)
Graphic x Flavor descriptor	-0.03 (0.08)	0.09 (0.08)	0.01 (0.08)	0.03 (0.10)
**Warning x Color**				
Text x Pink or purple pack	0.03 (0.06)	-0.02 (0.07)	-0.06 (0.07)	-0.01 (0.09)
Graphic x Pink or purple pack	0.03 (0.06)	-0.05 (0.07)	-0.08 (0.07)	0.07 (0.09)

^a^Main effects models include pack element variables and LCC use, adjusted for age, gender, race, ethnicity, sexual orientation, education, pre-existing perceptions of LCCs, and past 30-day use of tobacco products other than LCCs

^b^Pairwise contrasts were conducted between ever LCC users and current LCC users; after adjustment for multiple comparisons using the Tukey method, no significant differences were found between ever and current users for any of the four outcomes

^c^Interactions were estimated in separate models that included all variables shown in the table, adjusted for age, gender, race, ethnicity, sexual orientation, education, pre-existing perceptions of LCCs, and past 30-day use of tobacco products other than LCCs (all coefficients not shown)

^d^p < .05

^e^p < .01

^f^p < .001

### Interactions between pack elements

No interaction effects were found between cigarillo warning and flavor descriptor or between warning and pack color (Table 3). A significant interaction between LCC use and cigarillo flavor descriptor was observed for predicting flavor, taste, and appeal outcomes; specifically, flavor descriptors had less effect on current users’ perceptions of flavor and taste compared to never users (β = -0.22, p = .003; β = -0.22, p = .005, respectively), while flavor descriptors had a greater impact on current users’ ratings of product appeal (β = 0.20, p = .05). Further, flavor descriptors had less effect on ever users’ perceptions of product taste compared to never users (β = -0.17, p = .04).

## Discussion

To our knowledge, this study provides the first experimental evidence demonstrating that cigarillo packaging influences perceptions of cigarillos among young adult LCC users and non-users, supporting previous research on the influence of cigarette packaging in shaping consumer beliefs and consumption behavior [6–9,11–13,51]. Cigarillo pack flavor descriptors and colors significantly influenced product perceptions, and flavor descriptors, in particular, had a greater impact on how never users perceived product flavor and taste compared to current and ever users. While pack warnings decreased favorable perceptions of taste and smell, warnings did not moderate the effects of flavor descriptor or color.

These findings have several implications for broader limitations on cigarillo package labeling and design, as pack elements on these products can alter how consumers experience the product’s characteristics and their use of the product. Local, state, and national policymakers should consider bans on cigarillo packaging with overt flavor descriptors or imagery that connotates a flavor, or packaging that implies a certain flavor through generic descriptors such as “Tropical” [37]. Prohibiting non-overt flavor descriptors is particularly important, as the tobacco industry has been able to circumvent flavor bans by removing explicit flavor names while maintaining flavor chemicals in products [52]. Further, bans on flavor descriptors and associated flavor imagery could be accompanied by bans in actual product flavoring, similar to the flavor bans on cigarettes [53]. The flavor expectancies from product packaging, as well as the flavor chemicals that make products less harsh to smoke and give products an appealing taste and smell, are both influential factors in the perception, initiation, progression, and continuation of tobacco product use, particularly among youth and young adults [26,27].

These findings also support plain packaging regulations that might eliminate colors on cigarillo product packaging, if they imply a certain flavor or result in consumer misperceptions about the product (e.g., less harmful). Young adults in our study thought pink and purple packs were flavored and appealing—perceptions that were not overcome by pack warnings. Consumer behavior research suggests that consumers rely on common links between color and taste during visual processing of color cues on packaging [54–56], which influences perceptions of flavor attributes and product appeal [54,57,58]. It is well-documented that the tobacco industry uses pack colors and color descriptors to positively influence consumers’ perceptions of product characteristics [2,5], particularly after the terms “light”, “low”, or “mild” or similar descriptors on tobacco product packages were banned in the U.S. and elsewhere [8,10,12,59,60]. Color coding tobacco products through the use of color descriptors (e.g., “gold”, “silver”, “blue”) and associated pack color appear to have replaced prohibited descriptors, contributing to the perpetuation of consumer misperceptions about differential risk between products based solely on pack characteristics [8,61].

As the feasibility of removing colors from cigarillo and other tobacco product packaging in the U.S. remains uncertain, given the legal barriers and the litigious nature of the tobacco industry [62], further experimental studies in the U.S. are needed to build an evidence-base for plain packaging on diverse tobacco products that is robust to legal challenges. Examining the impact of plain packaging on behavioral outcomes—an area of research currently lacking [17]—as well as the role of pack colors on non-cigarette tobacco products is particularly important. Other regulatory action on pack colors in the U.S. could be taken in the meantime in relation to premarket review, a stipulation outlined in the Tobacco Control Act that requires tobacco companies to obtain authorization before legally marketing a new tobacco product [53]. Specifically, tobacco products with color changes to their packaging could be considered “new products” subject to rigorous product review (as pack color can alter how consumers perceive a product’s characteristics), including Social Science review that would evaluate the impact of the product on public health [5]. Although this process would not affect tobacco products that are already on the market, requiring premarket authorization of products with new or modified pack colors would involve a thorough product review by the FDA to ensure that any of these “new products” do not contain packaging elements that are false or misleading [5].

Limitations of this study include a web-based convenience sample, limiting the ability to generalize findings to all young adults in the U.S. While the sociodemographic characteristics of our sample differed slightly from the typical LCC user in the U.S., our study focused on young adults, the age group most likely to use LCCs [20,21]. Another limitation was the use of digitally derived images rather than actual representations of cigarillo packaging. While the lack of branding effect on product perceptions may be related to the use of a fictitious cigarillo brand, using a fictitious brand ensured participants were not influenced by pre-existing brand perceptions. Future research should explore whether warnings that cover a greater proportion of the pack (e.g., 50%) can overcome the effect of appealing pack elements and should also assess how packaging affects behavioral outcomes, including product initiation and use.

### Conclusions

Evidence that cigarillo packaging affects young adults’ product perceptions and does so in a manner that pack warnings may not overcome, provides novel insights into understanding how cigarillo packaging can influence cigarillo use. In addition to a ban on flavor descriptors (and their accompanying product flavoring), enacting stricter packaging regulations on LCCs (including restrictions on flavor cues and colors) could generate more negative responses toward such products and reduced product use.

## Supporting information

S1 File Dataset. (ZIP)

S1 Table Least square means for pack perceptions. (DOCX)

S2 Table Unadjusted mixed-effects model results for pack perceptions. (DOCX)
