# Supplementary material for: The effect of cigarillo packaging elements on young adult perceptions of product flavor, taste, smell, and appeal
Source: PLoS One. 2018 Apr 19;13(4):e0196236. doi: 10.1371/journal.pone.0196236 (PMC5909610; doi:10.1371/journal.pone.0196236)
Supplement: S1 Table — (DOCX) [file pone.0196236.s002.docx]

**S1 Table. Least Square Means for Pack Perceptions^a^**

| **Independent variables** | **Flavor** | **Taste** | **Smell** | **Appeal** |
| --- | --- | --- | --- | --- |
|  | **Least Square Mean (SE)** | **Least Square Mean (SE)** | **Least Square Mean (SE)** | **Least Square Mean (SE)** |
| **Models with only main effects^b^** | | | | |
| **Flavor descriptor** | | | | |
| None | 3.65 (0.03) | 4.02 (0.03) | 3.98 (0.03) | 3.57 (0.04) |
| Flavor descriptor | 4.01 (0.01) | 4.23 (0.02) | 4.12 (0.02) | 3.63 (0.02) |
| **Color** | | | | |
| No color | 3.77 (0.02) | 4.04 (0.03) | 3.97 (0.03) | 3.52 (0.04) |
| Pink or purple | 3.89 (0.02) | 4.21 (0.03) | 4.12 (0.02) | 3.69 (0.03) |
| **Type** | | | | |
| Box 5-pack | 3.81 (0.02) | 4.13 (0.02) | 4.04 (0.02) | 3.51 (0.03) |
| Foil 2-pack | 3.85 (0.02) | 4.12 (0.02) | 4.05 (0.02) | 3.70 (0.03) |
| **Branding** | | | | |
| No branding | 3.82 (0.02) | 4.12 (0.02) | 4.04 (0.02) | 3.60 (0.03) |
| Branded | 3.84 (0.02) | 4.13 (0.02) | 4.05 (0.02) | 3.61 (0.03) |
| **Warning** | | | | |
| No warning | 3.85 (0.02) | 4.17 (0.03) | 4.10 (0.03) | 3.62 (0.04) |
| Text-only | 3.81 (0.02) | 4.11 (0.03) | 4.02 (0.03) | 3.59 (0.04) |
| Pictorial | 3.83 (0.02) | 4.10 (0.03) | 4.01 (0.03) | 3.61 (0.04) |
| **LCC use** |  |  |  |  |
| Never | 3.76 (0.03) | 4.10 (0.03) | 3.96 (0.04) | 3.54 (0.05) |
| Ever | 3.90 (0.03) | 4.18 (0.03) | 4.13 (0.03) | 3.58 (0.04) |
| Current | 3.83 (0.03) | 4.10 (0.03) | 4.05 (0.03) | 3.69 (0.05) |
| **Models including interaction effects^c^** | | | | |
| **LCC use x Flavor descriptor** | | | | |
| Never user x No flavor descriptor | 3.49 (0.05) | 3.90 (0.05) | 3.83 (0.06) | 3.56 (0.07) |
| Never user x Flavor descriptor | 3.96 (0.03) | 4.23 (0.03) | 4.05 (0.04) | 3.55 (0.05) |
| Ever user x No flavor descriptor | 3.72 (0.05) | 4.11 (0.06) | 4.07 (0.06) | 3.62 (0.08) |
| Ever user x Flavor descriptor | 4.10 (0.03) | 4.28 (0.03) | 4.21 (0.03) | 3.62 (0.05) |
| Current user x No flavor descriptor | 3.73 (0.05) | 4.05 (0.05) | 4.02 (0.05) | 3.55 (0.07) |
| Current user x Flavor descriptor | 3.97 (0.03) | 4.18 (0.03) | 4.10 (0.03) | 3.74 (0.05) |
| **LCC use x Color** | | | | |
| Never user x No pack color | 3.67 (0.04) | 4.00 (0.05) | 3.88 (0.05) | 3.47 (0.07) |
| Never user x Pink or purple pack | 3.79 (0.03) | 4.13 (0.04) | 4.00 (0.04) | 3.64 (0.05) |
| Ever user x No pack color | 3.89 (0.04) | 4.11 (0.05) | 4.09 (0.05) | 3.59 (0.07) |
| Ever user x Pink or purple pack | 3.93 (0.03) | 4.27 (0.04) | 4.19 (0.04) | 3.65 (0.05) |
| Current user x No pack color | 3.77 (0.04) | 4.01 (0.04) | 3.95 (0.05) | 3.53 (0.06) |
| Current user x Pink or purple pack | 3.94 (0.04) | 4.22 (0.04) | 4.16 (0.04) | 3.76 (0.05) |
| **Warning x Flavor descriptor** | | | | |
| No warning x No flavor descriptor | 3.69 (0.05) | 4.11 (0.05) | 4.05 (0.05) | 3.62 (0.07) |
| No warning x Flavor descriptor | 4.03 (0.02) | 4.26 (0.03) | 4.16 (0.03) | 3.64 (0.04) |
| Text x No flavor descriptor | 3.56 (0.05) | 3.96 (0.05) | 3.89 (0.05) | 3.54 (0.07) |
| Text x Flavor descriptor | 4.00 (0.02) | 4.22 (0.03) | 4.11 (0.03) | 3.63 (0.04) |
| Graphic x No flavor descriptor | 3.68 (0.05) | 3.98 (0.05) | 3.98 (0.05) | 3.58 (0.07) |
| Graphic x Flavor descriptor | 4.00 (0.03) | 4.21 (0.03) | 4.09 (0.03) | 3.63 (0.04) |
| **Warning x Color** | | | | |
| No warning x No pack color | 3.82 (0.04) | 4.09 (0.04) | 4.01 (0.04) | 3.57 (0.06) |
| No warning x Pink or purple pack | 3.91 (0.03) | 4.28 (0.03) | 4.20 (0.03) | 3.70 (0.04) |
| Text x No pack color | 3.72 (0.04) | 4.00 (0.04) | 3.93 (0.04) | 3.52 (0.06) |
| Text x Pink or purple pack | 3.84 (0.03) | 4.18 (0.03) | 4.07 (0.03) | 3.65 (0.04) |
| Graphic x No pack color | 3.78 (0.04) | 4.03 (0.04) | 3.98 (0.04) | 3.50 (0.06) |
| Graphic x Pink or purple pack | 3.90 (0.03) | 4.17 (0.3) | 4.08 (0.03) | 3.71 (0.04) |

^a^Flavor ratings were assessed on a 5-point scale; taste, smell, and appeal ratings were assessed on a 7-point scale

^b^Main effects models include pack element variables and LCC use, adjusted for age, gender, race, ethnicity, sexual orientation, education, pre-existing perceptions of LCCs, and past 30-day use of tobacco products other than LCCs

^c^Interactions were estimated in separate models that included all variables shown in the table, adjusted for age, gender, race, ethnicity, sexual orientation, education, pre-existing perceptions of LCCs, and past 30-day use of tobacco products other than LCCs (all coefficients not shown)
