# Supplementary material for: The effect of cigarillo packaging elements on young adult perceptions of product flavor, taste, smell, and appeal
Source: PLoS One. 2018 Apr 19;13(4):e0196236. doi: 10.1371/journal.pone.0196236 (PMC5909610; doi:10.1371/journal.pone.0196236)
Supplement: S2 Table — (DOCX) [file pone.0196236.s003.docx]

**S2 Table.** **Unadjusted Mixed-Effects Model Results for Pack Perceptions**

| **Independent variables** | **Flavor** | **Taste** | **Smell** | **Appeal** |
| --- | --- | --- | --- | --- |
|  | **β (SE)** | **β (SE)** | **β (SE)** | **β (SE)** |
| **Control covariates** | | | | |
| **Age** | | | | |
| Age | 0.01 (0.01)^a^ | 0.02 (0.01)^b^ | 0.03 (0.01)^c^ | 0.00005 (0.01) |
| **Gender** | | | | |
| Male | Ref | Ref | Ref | Ref |
| Female | 0.09 (0.03)^c^ | 0.03 (0.03) | 0.03 (0.04) | -0.05 (0.05) |
| Other | -0.03 (0.11) | -0.32 (0.13)^a^ | -0.31 (0.14)^a^ | -0.11 (0.19) |
| **Race** | | | | |
| White | Ref | Ref | Ref | Ref |
| Black or African American | -0.11 (0.05)^a^ | -0.13 (0.06)^a^ | -0.04 (0.07) | 0.24 (0.09)^b^ |
| Asian | -0.08 (0.05) | -0.18 (0.06)^b^ | -0.14 (0.07)^a^ | 0.01 (0.09) |
| Other | -0.05 (0.05) | -0.14 (0.05)^a^ | -0.08 (0.06) | 0.06 (0.08) |
| **Ethnicity** | | | | |
| Non-Hispanic | Ref | Ref | Ref | Ref |
| Hispanic | -0.13 (0.04)^b^ | -0.17 (0.05)^c^ | -0.017 (0.06)^b^ | -0.12 (0.08) |
| **Sexual Orientation** | | | | |
| Heterosexual | Ref | Ref | Ref | Ref |
| Lesbian, gay, or bisexual | 0.11 (0.04)^b^ | -0.003 (0.04) | 0.02 (0.05) | -0.21 (0.06)^c^ |
| Other | 0.07 (0.08) | -0.15 (0.10) | -0.14 (0.11) | -0.47 (0.015)^b^ |
| **Education** | | | | |
| High school or less | -0.07 (0.04) | -0.06 (0.05) | -0.04 (0.05) | 0.20 (0.07)^b^ |
| Some college or associate’s degree | 0.02 (0.03) | 0.03 (0.03) | 0.02 (0.04) | 0.08 (0.05) |
| Bachelor’s degree or higher | Ref | Ref | Ref | Ref |
| **Pre-existing perceptions of LCCs** | | | | |
| Norms A | -0.03 (0.01)^a^ | 0.06 (0.02)^c^ | 0.10 (0.02)^c^ | 0.25 (0.02)^c^ |
| Norms B | 0.02 (0.01) | 0.11 (0.02)^c^ | 0.17 (0.02)^c^ | 0.27 (0.03)^c^ |
| Attitude | 0.02 (0.01)^a^ | 0.14 (0.01)^c^ | 0.18 (0.01)^c^ | 0.27 (0.02)^c^ |
| Risk of addiction | 0.04 (0.02)^a^ | -0.07 (0.02)^c^ | -0.12 (0.02)^c^ | -0.12 (0.03)^c^ |
| Risk of health problems | 0.05 (0.02)^a^ | -0.15 (0.02)^c^ | -0.20 (0.03)^c^ | -0.31 (0.04)^c^ |
| Prototype A | 0.05 (0.02)^b^ | 0.19 (0.02)^c^ | 0.24 (0.02)^c^ | 0.41 (0.03)^c^ |
| Prototype B | 0.02 (0.01) | 0.11 (0.01)^c^ | 0.15 (0.02)^c^ | 0.29 (0.02)^c^ |
| **Other tobacco use** | | | | |
| Past 30-day use of tobacco products other than LCCs | 0.04 (0.03) | 0.18 (0.03)^c^ | 0.26 (0.03)^c^ | 0.48 (0.05)^c^ |
| **Main covariates of interest** | | | | |
| **Flavor descriptor** | | | | |
| None | Ref | Ref | Ref | Ref |
| Flavor descriptor | 0.35 (0.03)^c^ | 0.20 (0.03)^c^ | 0.13 (0.03)^c^ | 0.06 (0.04) |
| **Color** | | | | |
| No color | Ref | Ref | Ref | Ref |
| Pink or purple | 0.11 (0.03)^c^ | 0.16 (0.03)^c^ | 0.14 (0.03)^c^ | 0.15 (0.04)^c^ |
| **Type** | | | | |
| Box 5-pack | Ref | Ref | Ref | Ref |
| Foil 2-pack | 0.04 (0.02) | -0.02 (0.03) | 0.001 (0.03) | 0.19 (0.03)^c^ |
| **Branding** | | | | |
| No branding | Ref | Ref | Ref | Ref |
| Branded | 0.01 (0.02) | 0.01 (0.03) | 0.02 (0.03) | 0.01 (0.03) |
| **Warning** | | | | |
| No warning | Ref | Ref | Ref | Ref |
| Text-only | -0.04 (0.03) | -0.05 (0.03) | -0.07 (0.03)^a^ | -0.02 (0.04) |
| Pictorial | -0.02 (0.03) | -0.07 (0.03)^a^ | -0.08 (0.03)^b^ | -0.01 (0.04) |
| **LCC use** |  |  |  |  |
| Never | Ref | Ref | Ref | Ref |
| Ever | 0.18 (0.03)^c^ | 0.25 (0.04)^c^ | 0.37 (0.04)^c^ | 0.28 (0.06)^c^ |
| Current | 0.09 (0.03)^b^ | 0.31 (0.04)^c^ | 0.46 (0.04)^c^ | 0.70 (0.06)^c^ |

^a^p<.05

^b^p<.01

^c^p<.001
